# The Change of Laboratory Tests Could Be Predictive Factors for Infection after McKeown Esophagogastrectomy

**DOI:** 10.1155/2019/9718705

**Published:** 2019-10-22

**Authors:** Chongxiang Chen, Tianmeng Wen, Qingyu Zhao

**Affiliations:** ^1^Department of Intensive Care Unit, Sun Yat-Sen University Cancer Center, State Key Laboratory of Oncology in South China, Collaborative Innovation Center for Cancer Medicine, Guangzhou, 510060, China; ^2^Guangzhou Institute of Respiratory Diseases, State Key Laboratory of Respiratory Disease, The First Affiliated Hospital of Guangzhou Medical University, Guangzhou, 510120, China; ^3^School of Public Health, Sun Yat-Sen University, Guangzhou, Guangdong Province, China

## Abstract

**Aim:**

To analyze whether the change of laboratory tests (postoperative day 1 (POD1) minus pre-operation) could be predictive factors for postoperative infection in patients who have undergone McKeown esophagogastrectomy.

**Methods:**

We retrospectively investigated the clinical data of 358 patients who have undergone McKeown esophagogastrectomy, and divided them into infection and noninfection groups. SPSS 22.0 software was performed for data analysis.

**Results:**

In the two groups, smoking status (66.7% vs. 42.3%; *P* = 0.014), male gender (86.1% vs. 72.0%; *P* < 0.001), hoarseness (23.6% vs. 8.7%; *P* < 0.001), poor coughing ability (51.4% vs. 9.1%; *P* < 0.001), the change of WBC count (5.59 ± 4.75 × 10^9^/L vs. 4.51 ± 4.11 × 10^9^/L; *P* = 0.05), the change of glucose (6.03 ± 3.97 g/L vs. 3.78 ± 3.18 g/L), the change of ALB (−12.83 ± 3.45 g/L vs. −10.69 ± 3.86 g/L), the change of CRE (0.17 ± 19.94 umol/L vs. −4.02 ± 15.40 umol/L, *P* = 0.047) were significantly different. These factors were assessed using logistic regression analysis, and factors with *P* ≤ 0.05 in the univariate analysis were entered into multivariate analysis based on the forward stepwise (conditional) method. Poor coughing ability (odds ratio [OR], 11.034, 95% confidence interval [CI], 5.358–22.724), smoking status (OR, 4.218; 95% CI, 2.110–8.431), the change of WBC count (OR, 1.079; 95% CI, 1.000–1.164), the change of serum ALB level (OR, 0.849; 95% CI, 0.772–0.935), and the change of blood glucose levels (OR, 1.237; 95% CI, 1.117–1.371) were determined as independent risk factors for postoperative infection. We established a scoring system based on these 5 factors, and the area under the curve for this predictive model was 0.843 (range, 0.793–0.894); the sensitivity, specificity, and cut-off score were 70.8%, 85.3%, and 2.500, respectively.

**Conclusion:**

Poor coughing ability, smoking habit, the high change of WBC and blood glucose levels, and low change of serum ALB levels can be used to predict the occurrence of postoperative infections among patients who have undergone McKeown esophagogastrectomy.

## 1. Introduction

Up to now, Esophageal cancer is the sixth-most common cause of cancer-related death all around the world, and in developing countries, it is the fifth most frequent cause of deaths [1]. Furthermore, the incidence and mortality of patients with esophageal cancer in China were the highest globally in 2009 [2]. Surgery remains the standard treatment for resectable esophageal cancer. However, esophagogastrectomy is a complex procedure, with morbidity and mortality rates of 23%–50% and 2%–8%, respectively [3, 4].

Patients undergoing McKeown esophagogastrectomy are exposed to a higher risk of infection compared with those receiving other types of surgery. Moreover, patients with esophageal cancer are at a greater risk of antimicrobial exposure due to their impaired immunological functions and are also at an increased risk of infection with multidrug-resistant bacteria.

In this study, we assumed that the change of laboratory tests (laboratory tests within 24 h after surgery minus pre-operation) will be associated with the infections following McKeown esophagogastrectomy, and developed recommendations for clinicians treating patients with these risk factors.

## 2. Methods

### 2.1. Data Collection

We collected clinical data from 358 esophageal cancer patients (including 268 male and 90 female patients) who were admitted for McKeown esophagogastrectomy (right thoracotomy followed by laparotomy and cervical anastomosis) between July 2014 and October 2016 at Sun Yat-Sen University Cancer Center (SYSUCC). The RDD number for this study is RDDA2019001127. The average age of the patients was 60.55 ± 7.87 years. We divided the patients into the infection and noninfection groups according to the occurrence of postoperative infections, and then retrospectively assessed the baseline characteristics, clinical disease features, perioperative features, preoperative, and postoperative laboratory test results (including white blood cell [WBC], neutrophil, hemoglobin [HB], aspartate aminotransferase [AST], alanine aminotransferase [ALT], serum albumin [ALB], blood urea nitrogen [BUN], creatinine [CRE], blood glucose, C-reactive protein [CRP], and lactic acid levels) between the groups. All the postoperative day 1 (POD1) indicators were analyzed within 24 h after surgery. And we used POD1 indicators minus preoperative ones to calculate the change of laboratory tests.

### 2.2. Inclusion and Exclusion Criteria

The inclusion criteria were as follows: patients aged >18 years with esophageal cancer who underwent McKeown esophagogastrectomy and developed an infection during hospitalization. And those with infection prior to hospital admission were excluded from the study.

### 2.3. Statistical Analysis

Categorical variables were expressed as number and percentage, and continuous variables were expressed as mean ± standard deviation. Student's *t*-test was used to examine continuous variables, and the Chi-squared test or Fisher's exact test was used to assess categorical variables. Multi-variate analysis was performed to determine the predictors of postoperative infection, and the forward stepwise (conditional) method was used to identify factors to enter into the multivariate regression model. Receiver operating characteristic (ROC) curves were constructed to estimate the sensitivity, specificity, and the area under the curve (AUC) for various cutoff points of the relevant indicators. Statistical significance was set at *P* ≤ 0.05, and all statistical analyses were computed using SPSS Version 22.0.

## 3. Results

### 3.1. Differences in the Baseline Characteristics

**Figure 1:**
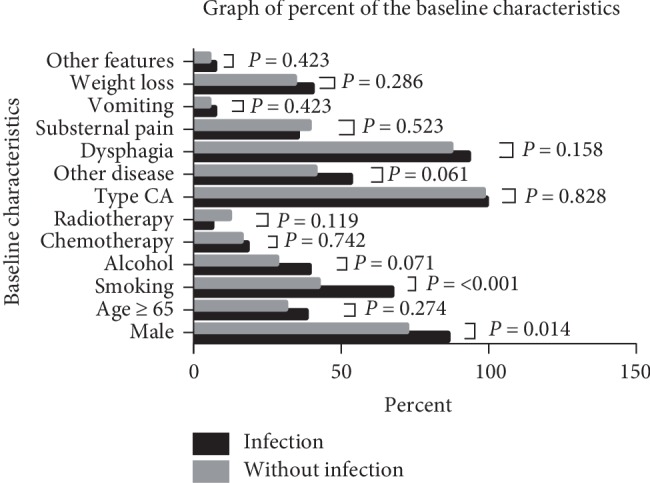
Baseline characteristics and clinical disease features between the infection group and noninfection group.

Table 1 describes the characteristics of the 72 patients (20.1%) with postoperative infection, from among the 358 patients who had undergone McKeown esophagogastrectomy in the present study. We compared the patients' baseline characteristics and clinical disease features between groups, and identified significant differences in smoking habits and gender between the two groups. The smoking habit frequency (66.7% vs. 42.3%; *P* < 0.001) and proportion of males (86.1% vs. 72.0%; *P* = 0.014) were greater in the infection group than in the noninfection group (Table 1 and Figure 1).

### 3.2. Differences in the Perioperative Clinical Features

**Figure 2:**
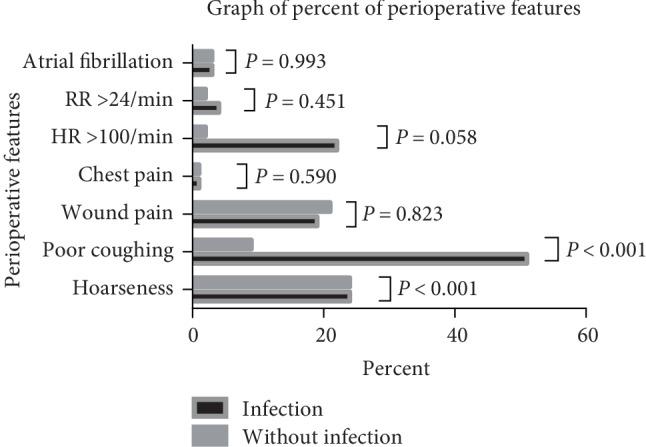
Difference in perioperative features among patients who underwent McKeon esophagogastrectomy.

In the present study, the factors of hoarseness (23.6% vs. 8.7%; *P* = 0.001) and poor coughing ability (51.4% vs. 9.1%; *P* < 0.001) were significantly different between the groups; both were more frequent in the infection group. However, other perioperative clinical features, including wound pain, increased heart rate and respiratory rate, chest pain/chest distress, and atrial fibrillation, did not exhibit a significant difference (Table 2 and Figure 2).

### 3.3. Differences in Change of Laboratory Test Results

**Figure 3:**
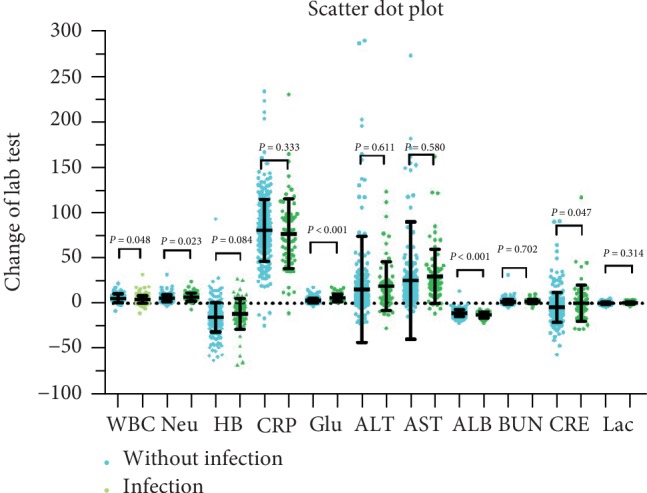
Scatter dot plot of the change of laboratory tests between two groups.

The results of the change of laboratory tests (POD1 minus pre-operation) were compared between the groups. The change of WBC count (5.59 ± 4.75×10^9^/L vs. 4.47 ± 4.14 × 10^9^/L; *P* = 0.048), neutropil count (7.00 ± 4.63 × 10^9^/L vs. 5.83 ± 3.69 × 10^9^/L; *P* = 0.023), glucose (6.03 ± 3.97 g/L vs. 3.78 ± 3.18 g/L; *P* < 0.001), ALB (−12.83 ± 3.45 g/L vs. −10.69 ± 3.86 g/L; *P* < 0.001), CRE (0.17 ± 19.94 umol/L vs. −4.02 ± 15.40 umol/L, *P* = 0.047) were greater in the infection group than in the noninfection group. None of the other change of laboratory test results showed significant differences (Table 3 and Figure 3).

### 3.4. Multivariate Analysis

Factors that were significant in the univariate analysis (*P* < 0.05) were included in the multivariate analysis. Accordingly, we assessed 5 factors, including poor coughing ability (odds ratio [OR], 11.034; 95% confidence interval CI, 5.358–22.724), smoking status (OR, 4.218; 95% CI, 2.110–8.431), the change of WBC count (OR, 1.079; 95% CI, 1.000–1.164), ALB level (OR, 0.849; 95% CI, 0.772–0.935), blood glucose level (OR, 1.237; 95% CI, 1.117–1.371), using multivariate regression; male gender and the other laboratory test results were not included (Table 4).

### 3.5. Development of a Scoring System to Predict Postoperative Infections

The AUC and cut-off point were 0.575 (range, 0.498–0.651) and 4.420 × 10^9^/L for the change of WBC count, 0.725 (range, 0.660–0.790) and 4.355 mmol/L for the change of blood glucose level, and 0.658 (range, 0.590–0.727) and −11.900 mmol/L for the change of serum ALB level, respectively.

Patients with were assigned a score of 1 for each of the following factors: poor coughing ability, smoking habit, the change of WBC count and blood glucose levels greater than the cut-off values, and the change of ALB level lower than the cut-off value; patients who did not meet these requirements were assigned a score of 0 each.

**Figure 4:**
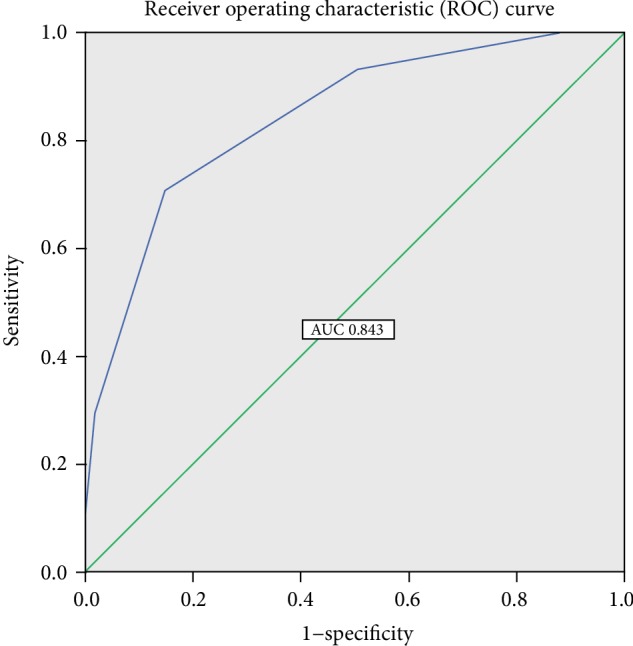
Receiver operating characteristic (ROC) curve of the scoring system.

The AUC of this predictive model was 0.843 (range, 0.793–0.894); the sensitivity, specificity, and cut-off score were 70.8%, 85.3%, and 2.500, respectively (Figure 4 and Table 5).

### 3.6. Pulmonary Complication through Clinical Diagnosis

The differences of changes of laboratory tests in the groups divided by pulmonary complication were showed in [Supplementary-material supplementary-material-1].

## 4. Discussion

Results of comparing the infection and noninfection groups in the present study indicated that poor coughing ability, smoking status, the change of WBC count, the change of ALB level, and the change of blood glucose level were independent risk factors for predicting postoperative infection in patients undergoing McKeown esophagogastrectomy.

According to our analysis, smoking acts as one of the independent risk factors for predicting postoperative infection. Liu et al. [5] reported that smoking history was one of the risk factors of postoperative lung infection. Moreover, the study conducted by Kinugasa et al.[6] showed that smoking habit was risk factors for postoperative pulmonary complications. Furtermore, the similar result shown in the study conducted by Ferguson et al. [7]. Smoking history could increase airway resistance, then cause numerous postoperative sputum thrombi. Consequently, the risk of pulmonary infection increases by impairment of the respiratory epithelium cilia structure, damaging to goblet cells, and weakening cilia movement.

The result of our study showed that the change of WBC count was associated with the postoperative infection. Sugita et al. [8] showed that the preoperative WBC count did not differ between infected and noninfected patients, although the POD1 WBC count was significantly higher in infected patients than in noninfected patients. The study conducted by Gomez et al. [9] also showed that the median WBC count was significantly greater in patients with infection than in those without infection during the first 10 postoperative days.

Furthermore, we found that the change of ALB level was an independent risk factor for postoperative infection in patients underwent McKeown esophagogastrectomy. The study conducted by Yin et al. [10] showed that low serum albumin was independently associated with the surgical site infections (SSI). Moreover, Zhao et al. [11] demonstrated that ALB level <35 g/L was an independent risk factor for postoperative infectious complications in patients with hepatocellular cancer. In addition, Yuwen et al. [12] showed that an ALB level of <35 g/L was associated with an increased risk of SSI in patients after orthopedic operations.

Lastly, our study showed that the change of blood glucose level was an independent risk factor for predicting infection. Similar to our findings, Vriesendorp et al. [13] indicated that the POD1 blood glucose level in esophageal cancer patients after esophagectomy was only associated with the length of hospitalization. Moreover, Ng et al. [14] showed that the change in the target glucose control in diabetic patients was independently associated with an increase in SSI. Another study conducted by Ambiru et al. [15] demonstrated that the SSI rates were correlated with the hyperglycemia following surgery.

In the scoring system of our study, the continuous variables changed to categorical variables through the cut-off value (higher than cut-off value is number A, lower than the cut-off value is number B), and added value for each factor. All the categorical variables used assigns 20% for each factor in the total score because it was useful and easy in clinical practice.

I think our study is quite novelty. On the one hand, our study talked about specific disease (esophageal cancer), and undergoing specific kind of operation (McKeown esophagogastrectomy); on the other hand, in our study, we used the changes of these laboratory tests different from other studies.

The limit of our study is that it is a single center retrospective research, and the study population comprised only Asian participants. The patient number enrolled in our study was relatively small, so some risk factor, such as alcohol consumption were not included in the independent risk factors. Probably, in the future study, we could get more data to conduct a tendentious matching analysis to better identify the risk factors. Moreover, we do not have postoperative pulmonary function tests in our clinical practice; we need prospective study to address this problem for better diagnosing the respiratory complication, which is one of the major complications of esophagectomy [16, 17]. Our study did not compare the minimally invasive esophagectomy with open invasive esophagectomy, some studies showed that minimally invasive esophagectomy had lower incidence of postoperative infection than open invasive esophagectomy [18–20]. In addition, other factors should be contained in further study, such as operation time, intraoperative bleeding, the application of antacids, and so on.

## 5. Conclusion

Patients are exposed to high risks of predicting postoperative infection after McKeown esophagogastrectomy, although poor coughing ability, smoking habit, the change of WBC count, the change of ALB level, and the change of blood glucose level may be as independent risk factors for postoperative infections in these patients. At last, we used a scoring system comprising these 5 factors, and observed that the AUC of this predictive model was 0.843 (range, 0.793–0.894), whereas the sensitivity, specificity, and cut-off score were 70.8%, 85.3%, and 2.500, respectively.

## Figures and Tables

**Table 1:** Baseline characteristics and clinical disease features between the infection group and noninfection group.

Outcome	Infection group (%)	Noninfection group (%)	*χ* ^2^	*P* value
*Sum*		286	—	
*Gender*			6.062	0.014^∗^
Male	62 (86.1)	206 (72.0)		
Female	10 (13.9)	80 (28.0)		
*Age*			1.195	0.274
≥65	27 (37.5)	88 (30.8)		
<65	45 (62.5)	198 (69.2)		
*Smoking habit*			13.695	<0.001^∗^
Yes	48 (66.7)	121 (42.3)		
No	24 (33.3)	165 (57.7)		
*Alcohol consumption*			3.254	0.071
Yes	28 (38.9)	80 (28.0)		
No	44 (61.1)	206 (72.0)		
*Chemotherapy*			0.108	0.742
Yes	13 (18.1)	47 (16.4)		
No	59 (81.9)	239 (83.6)		
*Radiotherapy*			2.431	0.119
Yes	4 (5.6)	34 (11.9)		
No	68 (94.4)	252 (88.1)		
*Type of cancer*			0.047	0.828
Squamous	71 (98.6)	281 (98.3)		
Others	1 (1.4)	5 (1.7)		
*Other chronic disease*			3.503	0.061
Yes	38 (52.8)	116 (40.6)		
No	34 (48.2)	170 (59.4)		
*Dysphagia*			1.995	0.158
Yes	67 (93.1)	249 (87.1)		
No	5 (6.9)	37 (12.9)		
*Substernal pain*			0.408	0.523
Yes	25 (34.7)	111 (38.8)		
No	47 (65.3)	175 (61.2)		
*Acid regurgitation/vomiting*			0.641	0.423
Yes	5 (6.9)	13 (4.5)		
No	67 (93.1)	273 (95.5)		
*Weight loss*			1.140	0.286
Yes	29 (40.3)	96 (33.6)		
No	43 (59.7)	190 (66.4)		
*Other clinical features*			0.641	0.423
Yes	5 (6.9)	13 (4.5)		
No	67 (93.1)	273 (95.5)		

^∗^Statistically significant at *P* ≤ 0.05.

**Table 2:** Difference in perioperative features among patients who underwent McKeon esophagogastrectomy.

Outcome	Infection group (%)	Noninfection group (%)	Statistic (*χ*^2^ or *T* value)	*P* value
Total no. of patients	72	286	—	
*Hoarseness*			12.282	<0.001^∗^
Yes	17 (23.6)	25 (8.7)		
No	55 (76.4)	261 (91.3)		
*Poor coughing ability*			70.967	<0.001^∗^
Yes	37 (51.4)	26 (9.1)		
No	35 (48.6)	260 (90.9)		
*Wound pain*			0.050	0.823
Yes	14 (19.4)	59 (20.6)		
No	58 (80.6)	227 (79.4)		
*Chest pain/chest distress*			0.290	0.590
Yes	1 (1.4)	2 (0.7)		
No	71 (98.6)	284 (99.3)		
*Heart rate*			3.586	0.058
<100/min	16 (22.2)	7 (2.4)		
≤100/min	56 (77.8)	279 (97.6)		
*Respiratory rate*			0.567	0.451
<24/min	3 (4.2)	7 (2.4)		
≤ 24/min	69 (95.8)	279 (97.6)		
*Atrial fibrillation*			0.000	0.993
Yes	2 (2.8)	8 (2.8)		
No	70 (97.2)	278 (97.2)		
*MAP*	90.20 ± 9.83	89.83 ± 10.33	−0.281	0.779

^∗^Statistically significant at *P* ≤ 0.05. MAP: Mean artery pressure

**Table 3:** Difference in the change laboratory test results between the infection and noninfection group (POD1 minus Pre-McKeown esophagogastrectomy).

	Infection group	Noninfection group	*T* value	*P* value
WBC (×10^9^/L)	5.59 ± 4.75	4.47 ± 4.14	−1.987	0.048^∗^
Neutrophils (×10^9^/L)	7.00 ± 4.63	5.83 ± 3.69	−2.276	0.023^∗^
HB (g/L)	−11.55 ± 17.01	−15.29 ± 16.21	−1.735	0.084
Serum ALB (g/L)	−12.83 ± 3.45	−10.69 ± 3.86	4.295	<0.001^∗^
ALT (IU/L)	19.18 ± 26.88	15.57 ± 58.71	−0.509	0.611
AST (IU/L)	29.72 ± 29.96	25.38 ± 64.76	−0.554	0.580
BUN (mmol/L)	2.06 ± 2.28	1.88 ± 2.77	−0.514	0.702
CRE (*μ*mol/L)	0.17 ± 19.94	−4.02 ± 15.40	−1.996	0.047^∗^
Glucose (mmol/L)	6.03 ± 3.97	3.78 ± 3.18	−4.525	<0.001^∗^
CRP (mg/L)	76.97 ± 38.48	81.21 ± 32.93	0.968	0.333
Lactic acid (mmol/L)	0.34 ± 1.32	0.18 ± 1.21	−1.007	0.314

^∗^Statistically significant at *P* ≤ 0.05. WBC, white blood cell; HB, hemoglobin; AST, aspartate aminotransferase; ALT, alanine aminotransferase; ALB, serum albumin; BUN, blood urea nitrogen; CRE, creatinine; CRP, C-reactive protein.

**Table 4:** Multivariate logistic regression analysis of the risk factors for infections after McKeown esophagogastrectomy.

	Univariate analysis			Multivariate analysis		
Variate	*P*	OR	95% CI	*P*	OR	95% CI
Gender	0.016	0.415	(0.203–0.850)			
Smoking habit	<0.001^∗^	2.727	(1.584–4.695)	<0.001^∗^	4.218	(2.110–8.431)
Poor coughing ability	<0.001^∗^	10.571	(5.725–19.520)	<0.001^∗^	11.034	(5.358–22.724)
Hoarseness	<0.001^∗^	3.227	(1.633–6.378)			
Change of ALB level	<0.001^∗^	0.855	(0.795–0.921)	=0.001^∗^	0.849	(0.772–0.935)
Change of WBC	0.051	1.059	(1.000–1.123)	=0.050	1.079	(1.000–1.164)
Change of neu	0.027	1.073	(1.008–1.143)			
Change of CRE	0.059	1.013	(1.000–1.027)	—	—	—
Change of glucose	<0.001^∗^	1.283	(1.177–1.398)	<0.001^∗^	1.237	(1.117–1.371)

^∗^Statistically significant at *P* ≤ 0.05. Change of ALB: serum ALB level within 24 h after surgery minus pre-operation. Change of WBC level: serum WBC level within 24 h after surgery minus pre-operation. Change of neutropils: serum neutropils level within 24 h after surgery minus pre-operation. Change of CRE: serum CRE level within 24 h after surgery minus pre-operation. Change of glucose: blood glucose level within 24 h after surgery minus pre-operation. Factors were entered into multivariate regression using the forward stepwise (conditional) approach (*P* ≤ 0.05).

**Table 5:** Receiver operating characteristics of the independent risk factors and the scoring system.

Factors	AUC (95% CI)	*P* value	Cut-off	Sensitivity (%)	Specificity (%)
Change of WBC level	0.575 (0.498–0.651)	0.039^∗^	4.420	61.1	53.8
Change of glucose level	0.725 (0.660–0.790)	<0.001^∗^	4.355	62.5	74.8
Change of ALB level	0.658 (0.590–0.727)	<0.001^∗^	−11.900	59.7	67.1
Scoring system	0.843 (0.793–0.894)	<0.001^∗^	2.5	70.8%	85.3%

^∗^Statistically significant at *P* ≤ 0.05. Change of ALB: serum ALB level within 24 h after surgery minus pre-operation. Change of WBC level: serum WBC level within 24 h after surgery minus pre-operation. Change of glucose: blood glucose level within 24 h after surgery minus pre-operation. With regard to the scoring system, patients were assigned a score of 1 for each of the following factors: poor coughing ability, smoking habit, change of WBC count and blood glucose levels greater than the cut-off values, and change of ALB level lower than the cut-off value, whereas patients who did not meet these requirements were assigned a score of 0 each.

## Data Availability

The datasets used and/or analyzed in the current study are available from the corresponding author upon request.

## Supplementary Materials

Supplementary Materials The supplementary data includes a table of the difference of the changes of laboratory tests divided by clinical diagnosis pulmonary complication.
