# Supplementary material for: The Change of Laboratory Tests Could Be Predictive Factors for Infection after McKeown Esophagogastrectomy
Source: Biomed Res Int. 2019 Oct 22;2019:9718705. doi: 10.1155/2019/9718705 (PMC6854965; doi:10.1155/2019/9718705)
Supplement: Supplementary Materials — The supplementary data includes a table of the difference of the changes of laboratory tests divided by clinical diagnosis pulmonary complication. [file 9718705.f1.pdf]

The change of laboratory tests could be predictive factors for infection after McKeown esophagogastrectomy

Chongxiang Chen[1], Tianmeng Wen[2], Qingyu Zhao[1#]

[1]Department of Intensive Care Unit, Sun Yat-sen University Cancer Center; State Key Laboratory of Oncology in South China; Collaborative Innovation Center for Cancer Medicine, Guangzhou 510060, China;

[2] School of Public Health, Sun Yat-sen University, Guangzhou, Guangdong Province, China

Chongxiang Chen: 599232426@qq.com

Tianmeng Wen: wentianmeng163@163.com

Qingyu Zhao: zhaoqy163@163.com

# Correspondence：

Address

Tel/Fax 86-13808896565

E-mail: zhaoqy163@163.com

Supplementary Table. Clinical diagnosis pulmonary complication

	Pulmonary Complication	Non-Pulmonary	T value	P value

	Group (77)	complication Group (281)		
**WBC (×10⁹/L)**	5.57±5.65	4.45±3.81	-1.643	0.104
**Neutrophils (×10⁹/L)**	7.01±5.40	5.80±3.37	-1.863	0.066
**HB (g/L)**	-11.89±17.62	-15.26±16.03	-1.603	0.110
**Serum ALB (g/L)**	-12.33±3.48	-10.79±3.91	3.131	0.002*
**ALT (IU/L)**	24.85±38.05	13.95±57.23	-1.577	0.116
**AST (IU/L)**	37.07±48.18	23.28±61.88	-1.810	0.071
**BUN (mmol/L)**	2.25±2.26	1.83±2.78	-1.231	0.219
**CRE (μmol/L)**	0.10±20.00	-4.34±16.51	-1.995	0.047*
**Glucose (mmol/L)**	5.36±4.17	3.42±2.71	-3.865	<0.001*
**CRP (mg/L)**	75.31±37.10	81.24±34.52	1.315	0.189
**Lactic acid (mmol/L)**	0.48±1.29	0.12±1.26	-2.175	0.030*
